# Neoproterozoic copper cycling, and the rise of metazoans

**DOI:** 10.1038/s41598-019-40484-y

**Published:** 2019-03-06

**Authors:** J. Parnell, A. J. Boyce

**Affiliations:** 1 School of Geosciences, University of Aberdeen, Aberdeen, AB24 3UE United Kingdom; 2 Scottish Universities Environmental Research Centre, Glasgow, G75 0QF United Kingdom

## Abstract

The rise of animal life is temporally related to the increased availability of oxygen in the hydrosphere and atmosphere during the Neoproterozoic. However, the earliest metazoans probably needed relatively low oxygen concentrations, suggesting additional environmental and/or biochemical developments were involved. Copper was required in the exploitation of oxygen by the evolving animals, through the development of respiratory proteins and the extracellular matrix required for structural support. We synthesize global data demonstrating a marked enrichment of copper in the Earth’s crust that coincided with the biological use of oxygen, and this new biological use of copper. The copper enrichment was likely recycled into the surface environment by weathering of basalt and other magmatic rocks, at copper liberation rates up to 300 times that of typical granitic terrain. The weathering of basalts also triggered the Sturtian glaciation, which accelerated erosion. We postulate that the coincidence of a high availability of copper, along with increased oxygen levels, for the first time during the Neoproterozoic supported the critical advances of respiration and structural support in evolving animals.

## Introduction

The precise timing of the first metazoans is uncertain, but the evidence focuses on a time at or shortly after the first of the two major Neoproterozoic glacial intervals (‘Sturtian’) as the time at which metazoans started to appear^1,2^. This was a time of increasing concentrations of oxygen in the atmosphere, inviting inference of a genetic link between the availability of oxygen and animal evolution^3–6^. However, recent assessments of the requirements of the first metazoans conclude that oxygen was only needed at low levels that had already been available for some time^5–8^. This implies that a Neoproterozoic oxygenation event was not the sole requirement for the rise of animal life. The use of oxygen required availability of copper^9–11^, which we show was likely being cycled at unprecedented levels in surface environments at that time. Thus, the higher oxygen levels of the late Neoproterozoic were exploited by innovative use of the available copper.

**Figure 1:**
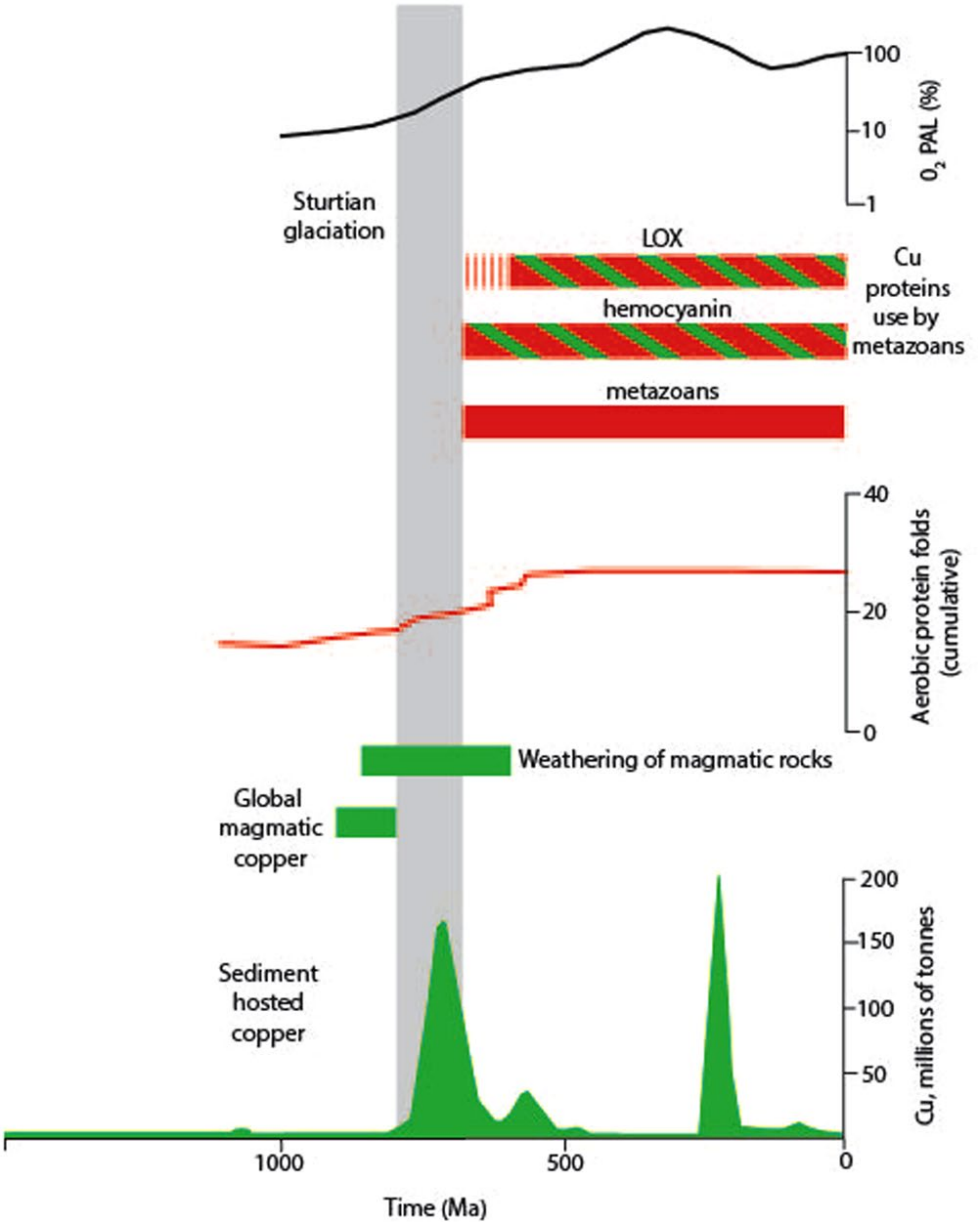
Timeframe for magmatic and sedimentary copper mineralization, enhanced weathering of magmatic rocks, metazoan evolution, and copper protein utilization. Data from refs^22,23,29,31,32,40^. Oxygenation profile from ref. ^4^. Copper concentrations also exhibit high levels in Neoproterozoic black shales, shown by Cu/Ti levels^47^ and copper in pyrite^48^. First use of copper proteins by metazoans uncertain but at least as old as shown. PAL = Present Atmosphere Level.

Copper is used by all domains of life^12–14^, and it has been inferred that the availability of copper influenced the timing of the evolution of multicellular life^15,16^. Proteins evolved to utilize copper in new ways following the oxygenation of the Earth^13,17,18^. The new copper proteins were, accordingly, used by animals and other eukaryotes (Fig. 1). Copper is used by organisms living in oxygen-rich environments, while most anaerobes do not use it^13^. There was a marked increase in protein fold domains (biologically functional 3-D structures) related to aerobic metabolism at ~700 Ma^17^, i.e. at about the time of the Sturtian glaciation, and today up to 160 copper proteins are found in eukaryotes^12^ Initially, copper proteins helped to bind and neutralize oxygen and negate oxygen toxicity^9,10^, but copper also conferred benefits to enhance the radiation of metazoans. Cupredoxins catalysed the reduction of oxygen to water in the respiratory chain^18,19^. The copper-bearing cytochrome c oxidase (COX) is the terminal enzyme in the mitochondrial respiratory chain required for the synthesis of ATP for energy in cells^20^.Two classes of copper protein are of particular importance to animals in allowing the breathing of oxygen, and the development of body architecture. These are the oxygen-carrying hemocyanin respiratory proteins, and lysyl-oxidases (LOX) whose primary role is in the modelling of extracellular matrix, including the synthesis of collagen and elastin^11,21^ essential to the transition from unicellular to multicellular organisms, including animals. These copper-dependent proteins diversified in the Neoproterozoic, coincident with the flourishing of animals^14,22^.

Evidence for the antiquity of hemocyanin in molluscs derives from a ~735 Ma molecular clock age^23^ and an inferred occurrence in the Cambrian Burgess Shale^24^. This is consistent with a general increase in the biological use of copper at that time^15^. Sponges are the earliest diverging metazoan group, for which body fossils, molecular clock dates and lipid biomarkers all indicate a record back to the mid-Cryogenian, following the Sturtian glaciation^3,25,26^. The copper protein hemocyanin was used by the last common metazoan ancestor and accordingly, was used by the earliest sponges^27^. Sponges were similarly the first organisms in which LOX enzymatic activity may have sculpted extracellular matrix to allow, for example, structural support^22^. The most abundant protein in animals is collagen, which provides essential mechanical support and was used by the earliest metazoans^28,29^. A requirement for oxygen in collagen has formerly been viewed as an expression of how animal evolution was dependent upon oxygen^30^, but recently this constraint has been questioned^5^. An alternative essential requirement for collagen synthesis is copper^11,21^, based on the LOX enzymes. Collagen formation may have been a consequence of the detoxification of oxygen^31^, which copper proteins engendered^9,10^. The exploitation of these copper-dependent proteins was therefore fundamental to the earliest metazoan physiology.

Whilst copper had always been available, albeit in lower concentrations^15^, the increased levels of oxygen in combination with anomalous availability of copper allowed a major innovation in how copper was used. There had even been previous episodes of high copper availability^32^, but before the critical rise in oxygen. This coincidental availability of relatively high levels of both copper and oxygen allowed their integrated use by the new metazoans. Further, the increased oxygen levels also ensured that the copper could be readily recycled at the Earth’s surface. Copper is strongly redox-sensitive, and in oxidizing conditions is mobile^33^. Thus, the high oxygen facilitated high copper mobility and availability, which in turn could be used by the metazoans to exploit the oxygen. The focus on copper as the element important to the metazoans reflects the critical combination of its abundance in basalts and mobility in oxidizing conditions, which is a combination not shared by other elements.

The Sturtian glaciation at ~700 Ma likely enhanced the availability of copper to a possible role in subsequent evolution. Glacial erosion is especially important as a source of finely ground material with high bioavailability^34^. The global Sturtian event in particular caused extensive deep erosion of copper-enriched crust, bringing copper to the surface environment just as oxygen levels increased and the first metazoans appeared. The Proterozoic crust had become enriched in copper^32^ through a succession of Palaeoproterozoic-Mesoproterozoic volcanic massive sulphide (VMS), copper porphyry and other granite-hosted deposits. Then, global Neoproterozoic magmatic activity^35^ was widely associated with copper mineralization. Volcanic deposits on at least thirteen palaeocontinents host copper mineralization in the 900 to 700 Ma interval, prior to the Sturtian glaciation (Table 1).

**Table 1:** Global occurrence of Early Neoproterozoic Copper ore, and ore grades in volcanic rocks, in 13 tectonic plates.

Plate	Locality	Age (Ma)	Cu ore (%) (Ref.)	Volcanic setting
Laurentia	Victoria Island, NWT	~725	<0.1-> 4.0^55^	basalts
South China	Pingshui	~900	1.03^56^	volcanic massive sulphide
North China	Jinchuan	~825	0.7^57,58^	rift-related magmatism
Siberia	Ioko-Dovyren	740–700	0.28–0.64^59^	rift-related mafic intrusion
India	Khetri Copper belt	~850	1.1–1.7^60^	rift-related volcanics
Arabia	Saudi Arabia	~800	0.37–2.5^61^	volcanic massive sulphide
Nubia	Eritrea	~780	0.99–3.91^62^	volcanic massive sulphide
West Africa	Morocco	750–700	2.5–3.5^63,64^	rift-related volcanics
Congo	N. Namibia	~745	1–10^65^	rift-related volcanics
Kalahari	S. Namibia	900–800	3^66^	rift-related volcanics
Sao Francisco	Mara Rosa Arc	900–800	0.43^67,68^	volcanic arc Cu-Au
Rio de la Plata	Uruguay	~715	?^69^	volcanic massive sulphide
Australia	South Australia	~800	~3^70^	syn-sedimentary magmatism

**Figure 2:**
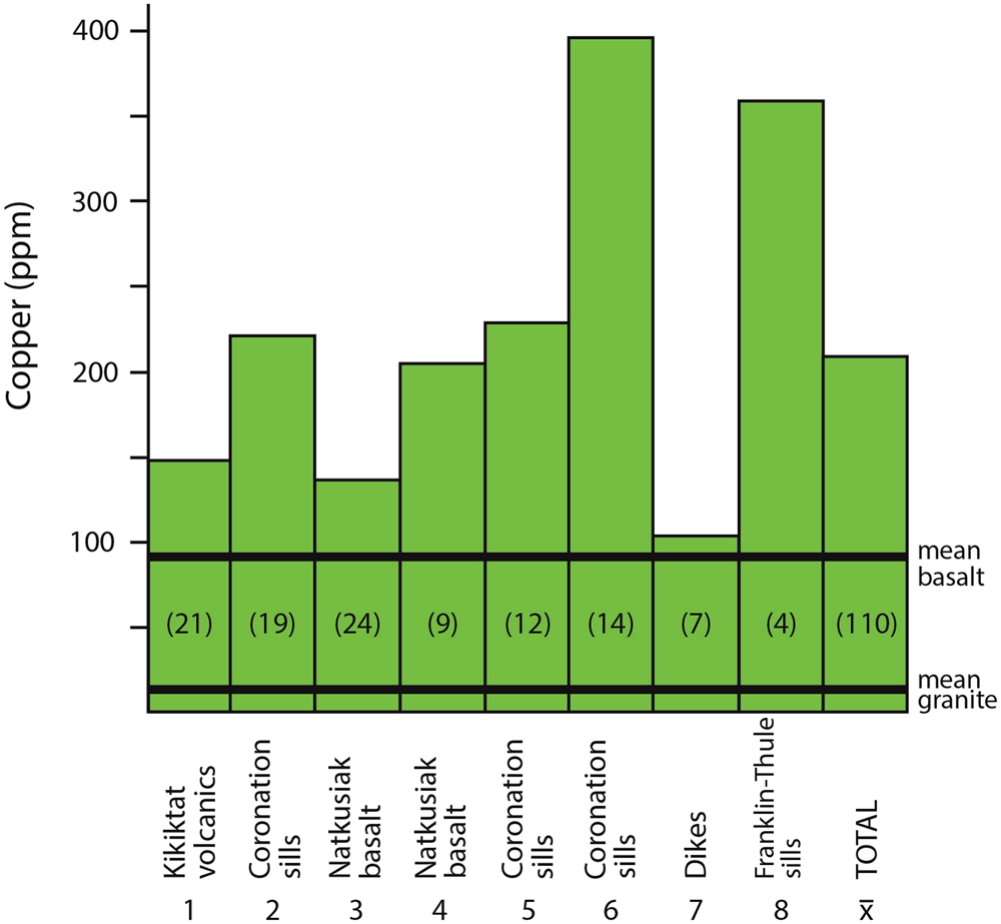
Anomalous copper contents in Franklin Igneous Province. Copper contents for 8 sets of samples in FIP, and mean value, relative to global mean values for basalt and granite. Data sources in refs ^71–76^ (Table 2).

Weathering of the Neoproterozoic basalts has been implicated in carbon dioxide drawdown and cooling, which triggered the Sturtian glaciation^6,36–38^. The basalts of this magmatic episode would have been highly weatherable^6,36^, and the weathering of an atypically large volume of basalt at that time would have liberated anomalous amounts of copper to surface systems. The average basalt has a copper content about seven times that of the average granite (mean values 90 ppm basalt, 13 ppm granite^39^), which would normally have dominated the detritus from eroding continents. Considering also the greater susceptibility of basalt to weathering, up to twenty times faster than granitic rocks^40^, the erosion of typical basaltic terrain might increase the flux of copper by two orders of magnitude relative to typical granitic terrain. The contrast would have been even greater in the Cryogenian, when the basalts were conspicuously mineralized by copper. Even unmineralized early Cryogenian basalts contain copper levels higher than normal. The model for accelerated weathering is based on basalts on the Laurentian continent^8,38,40^, where the Franklin Igneous Province (FIP) covers an area exceeding 2 million km^2^. Multiple data sets from Alaska to Greenland show mean copper levels in the FIP over twice those of average basalts (Fig. 2, Table 2), and FIP basalts also contain native copper. These statistics combine to indicate the liberation of copper by weathering of the FIP up to 300 times that of granitic terrain, independent of any additional enhancement due to rapid glacial weathering. Evidence from ɛNd values for an enhanced contribution of eroded magmatic rock to marine sediments from about 750 to 600 Ma^40^ suggests that this potential was realized.

**Table 2:** Copper contents in basalts, Laurentia.

Region		Unit	Age (Ma)	Cu content (%) (n)	Reference
1.	Alaska	Kikiktat volcanics	719.5	146.7 (21)	^71^
2.	Nunavut	Coronation sills	723	220.4 (19)	^72^
3.	Victoria Island	Natkusiak Fm. Basalt	723	135.6 (24)	^73^
4.	Victoria Island	Natkusiak Fm. basalt	723	210.0 (9)	^74^
5.	Murray Island	Coronation sills	723	228.3 (12)	^75^
6.	Bathurst Inlet	Coronation sills	723	396.4 (14)	^75^
7.	Somerset/POW Islands	Dikes	723	101.0 (7)	^75^
8.	Greenland	Franklin-Thule sills	716–720	357.5 (4)	^76^

The high flux of copper into the surface/near-surface environment immediately before the Sturtian glaciation is evident from the widespread copper mineralization of clastic sedimentary successions beneath the diamictite. It is very likely that these copper reservoirs were exposed during the extensive (global, long-lived) Cryogenian glaciation, which would have caused further weathering and erosion. Where glacial diamictites are preserved, there is no doubt about the supply of copper. Sturtian diamictites lie unconformably on copper-mineralized rocks in Australia, Africa, North America and Greenland. Clasts of copper ore in the diamictites, in Canada^41^ and Greenland^42^, convincingly demonstrate down-cutting into the underlying ores. FIP basalts interfinger with the diamictite in Alaska and contribute clasts to it^43^. Evidence of copper sulphide replacement of early pyrite in the Central African Copper Belt indicate that copper mineralization was still taking place during burial of the diamictites^44^. Similarly, copper mineralization in south Australia continued from pre-diamictite volcanic rocks into post-diamictite sediments^45^, indicating that copper cycling spanned the period of glaciation, and also left ore in periglacial breccias^46^. The African copper belt deposits, together with early Cryogenian copper deposits in western Canada, constitute most of the known resources of sediment-hosted copper^32^, implying the availability of exceptional amounts of copper in the upper crust at that time. In summary, a widespread copper-rich substrate to the diamictites, copper-rich basalt interfingering with diamictite, copper-rich detritus in diamictites, and the flush of copper-rich fluids through diamictites combine to indicate an unprecedented flux of copper to the Sturtian surface.

Globally, the enhanced availability of copper in the surface environment is evident from the chemistry of marine organic-rich shales^47^ and the chemistry of pyrite^48^ precipitated during the early burial of the shales, which give a measure of seawater composition. Copper levels in Neoproterozoic shales and the diagenetic pyrite are both higher than at any other phase of Earth’s history^47,48^. This records a higher supply of copper than hitherto, which was extracted from seawater by the precipitation of sulphides under locally anoxic conditions. Following the Neoproterozoic, as the oceans became predominantly oxic, more copper was retained in solution and thereby bioavailable. The surface chemistry in the immediate aftermath of the Sturtian glaciation can be inferred from the earliest diagenetic sulphides in the postglacial succession. The sulphur isotope composition of diagenetic pyrite in the glacial-postglacial succession indicates an origin through (low-temperature) microbial sulphate reduction^49^. Exceptionally, the postglacial sulphides include copper sulphides, with isotopic compositions similar to those of accompanying pyrite, and also attributable to microbial sulphate reduction^49^. The formation of discrete copper sulphides is strong evidence for a copper-rich environment during and after the glaciation^49^.

It is possible that episodes of copper mineralization are missing from the earlier Proterozoic record due to gaps in preservation, but the occurrence of multiple examples of both magmatic and sedimentary copper mineralization in the Neoproterozoic strongly suggests that this was an exceptional period of copper delivery to the upper crust. Basalts in general are commonly mineralized by copper, to the extent that they constitute a recognized ore type^50^, so the huge FIP represented a major reservoir of copper. The release of copper from basalts was enhanced under oxic conditions^51^. The anomalous availability of copper during the Sturtian glaciation does not mean that the glaciation was critical to the flux of copper. Rather, the glaciation and release of copper were both products of a single process, i.e. the weathering of the FIP basalts, which consumed carbon dioxide to cause cooling^36^ and liberated the metal. This is why the sediments below the Sturtian diamictite were already mineralized by copper-rich groundwaters in several parts of the world. The model for weathering-induced cooling emphasizes that weathering was enhanced shortly before the glaciation due to a combination of continental break-up and low latitude position of the continents^36,37,40^. Break-up triggered magmatism, and an equatorial setting provided a suitable tropical climate in which basalts yield a large proportion (>50%) of their copper^52^. Thus these factors would have accelerated the flux of copper to surface environments prior to, and during, the Sturtian glaciation.

**Figure 3:**
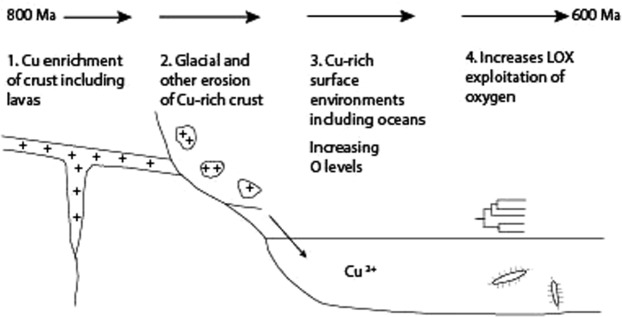
Schematic cycling of copper in Neoproterozoic era. Early Neoproterozoic enrichment of the crust in copper, followed by glacial and other erosion, introduced copper to surface environments where it facilitated use of elevated oxygen by metazoans.

The high availability of copper was not a short-lived phenomenon. Following the Sturtian glaciation, the record of high copper flux continued in the later Cryogenian and Ediacaran, including for example copper-mineralized flood basalts across Eastern Europe and Ukraine^53^, and mineralized sediments in North Africa, Siberia and Australia^45,54^. The continuing record of copper mineralization, and recycling of older Proterozoic copper deposits, in the latter part of the Neoproterozoic, shows that the anomalous supply of copper persisted from the origin of the metazoans through to the Cambrian explosion of life. Following peak delivery of copper to the upper crust, continued access to the copper was facilitated by the higher oxygen content of the atmosphere from the late Neoproterozoic onwards. Increased oxygen allowed greater concentrations of dissolved copper in surface waters and seawater^33^. Prior to the late Neoproterozoic, when oceans were anoxic, lower levels of dissolved copper limited its potential use. Then in the late Neoproterozoic copper was readily available to support the development of the copper-dependent proteins that facilitated the early evolution and diversification of animals (Fig. 3).

## Methods

Copper contents in basalts in the Franklin province of the Laurentian continent were collated from published literature, and summarized as mean values for distinct regions. The composite value for the whole province is a weighted mean from constituent regions. Copper ore grades are most recent estimates available for deposits considered viable for mining, in published literature. Estimates are for either the largest deposit or ore province in each continent.

## Supplementary information

Supplementary Information
