# Supplementary material for: Neoproterozoic copper cycling, and the rise of metazoans
Source: Sci Rep. 2019 Mar 6;9:3638. doi: 10.1038/s41598-019-40484-y (PMC6403403; doi:10.1038/s41598-019-40484-y)

**Neoproterozoic copper cycling, and the rise of metazoans**

J. Parnell & A. J. Boyce

**Supplementary Materials**

**Fig. S1. Compilation of Cu contents in pyrite in black shale, for 200 Myr intervals. Determined by LA-ICP-MS, in ref. 48. Data for 600-400 Myr also shown separated into latest Precambrian/Cambrian and Ordovician contributions, to highlight progressive decline in mean Cu content for Neoproterozoic.**


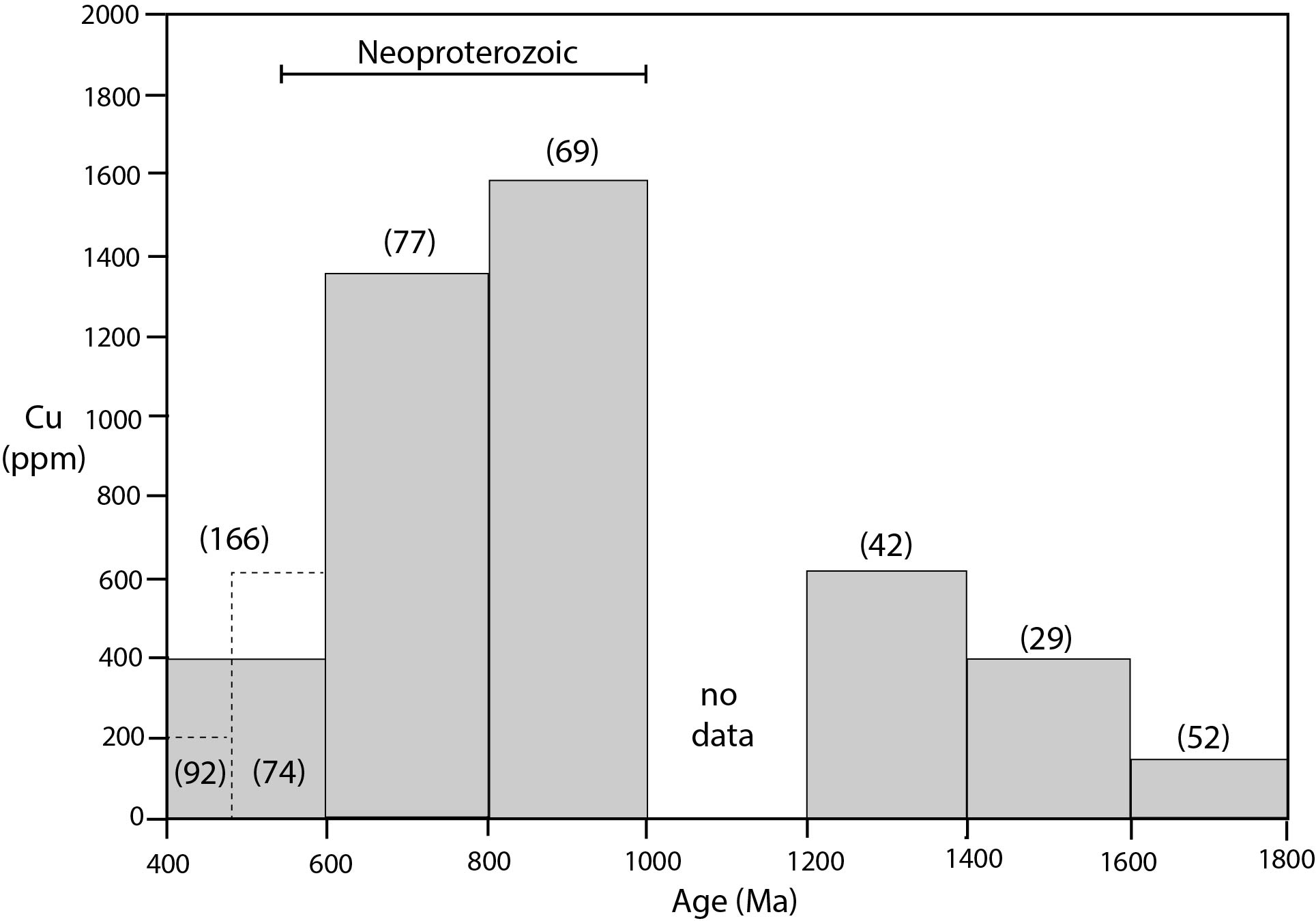

Supplement: Supplementary file 1 — Supplementary Information [file 41598_2019_40484_MOESM1_ESM.docx]
